# Effects of victim’s body posture and attacker’s gender on slashing attacks: a biomechanical study

**DOI:** 10.3389/fbioe.2024.1450953

**Published:** 2024-09-03

**Authors:** Shangxiao Li, Shufang Yuan, Yi Shi, Bin Ni, Wenxia Guo, Chaopeng Yang, Mingzhi Wang, Weiya Hao

**Affiliations:** ^1^ Research Center for Sports Psychology and Biomechanics, China Institute of Sport Science, Beijing, China; ^2^ Department of Health Service, Logistics University of People’s Armed Police Force, Tianjin, China; ^3^ Institute of Forensic Science, The Ministry of Public Security of the People’s Republic of China, Beijing, China

**Keywords:** forensic biomechanics, slashing attack, motion analysis, sharp force injuries, crime prevention

## Abstract

**Objective:**

Sharp force injury has been and will remain to be a major cause of violent death; however, scientific evaluations on the impact of body posture of the victim and gender of the perpetrator on sharp force injury have been scarce. The purpose of this study was to evaluate the biomechanical characteristics found in individuals (male and female) when using a Chinese kitchen knife to slash the neck of a dummy while it was in the standing and supine positions. This work offers a solid basis for forensic identifications, criminal investigations, and court trials.

**Methods:**

A total of 12 male and 12 female college students participated in this study. Kinematic, kinetic, and surface electromyography (sEMG) data were evaluated when slashing the neck of a dummy while it was in the standing and supine positions using a Chinese kitchen knife.

**Results:**

When slashing the neck of a standing dummy, participants showed shorter contact time (19.5%) and slower shoulder velocities (30.9%) as well as higher hip velocity (26.0%) and increased root mean square (RMS) and integral electromyography (iEMG) for the anterior deltoid (51.3% and 51.2%, respectively) compared to those while the dummy was in the supine position (all *p* < 0.05), regardless of gender. When slashing a dummy’s neck while it was in standing and supine positions, male participants showed higher shoulder, elbow, and wrist velocities (22.6%, 22.7%, and 24.4%, respectively) and higher slashing velocity (19.8%), slashing force (24.2%), and energy (46.2%) than female participants (all *p* < 0.05). In addition, male participants showed shorter contact time (17.8%), and the values of RMS and iEMG of the anterior deltoid, biceps brachii, extensor carpi radialis longus, and flexor carpi ulnaris were less than those of female participants (98.9%, 47.3%, 65.6%, and 33.4% for RMS and 115.1%, 59.4%, 80.1%, and 47.8% for iEMG, respectively).

**Conclusion:**

There was no difference in slashing speed, slashing force, and energy when using a Chinese kitchen knife to slash the dummy’s neck while it was in different body postures (standing and supine), suggesting a similar level of injury severity. However, there were significant differences in slashing action patterns between the two body postures, with longer contact time, smaller hip velocity, greater shoulder velocity, and less muscle activation level of the deltoid exertion when slashing the dummy’s neck in the supine position. Gender may have a greater effect on the severity of slashing, and the gender difference may be partly related to the body weight difference. The findings from this study may provide quantitative indicators and references for analyzing the motive behind the crime, as well as for case reconstruction, and for the court’s conviction and sentencing processes.

## Introduction

Sharp force injury has been and will remain to be a major cause of violent death (Kristoffersen et al., 2016; Carmichael et al., 2018). Sharp instruments were used in up to 80% of all homicidal cases (Park and Son, 2017; He et al., 2014; Khelil et al., 2018), especially in some countries where use of firearms is strictly controlled. The proportion of sharp force injuries committed by men is significantly higher than that committed by women (Christensen et al., 2016; Kristoffersen et al., 2014). The perpetrators are predominantly young, with the majority aged between 18 and 44 (Wu et al., 2019; Christensen et al., 2016; Kristoffersen et al., 2014).

Knives, axes, scissors, daggers, awls, bayonets, and sharp glass are common sharp instruments, of which knives are the most commonly used weapon (Park and Son, 2017; Kidd et al., 2014). Sharp force injury is mainly stabbing if the radius of the knife tip is sharp, while the injury is slashing injury if the radius of the knife tip is large. In China, the most accessible sharp instrument is the kitchen knife (Wu et al., 2019), and the knife’s blade is rectangular with no tip. Therefore, the main damage caused by Chinese kitchen knives is through slashing.

The neck is the most common place where perpetrators attack with weapons (Kemal et al., 2013; De-Giorgio et al., 2015). There are important anatomical structures, such as the trachea and carotid artery, in this area. Injuries from slashing on the neck may cause difficulty breathing or acute hemorrhage shock, which could be fatal. When the victim is attacked in the supine position (such as drunk and asleep), the incident is often a premeditated crime, meticulously planned in advance. When the victim is attacked when he/she is in a standing position, the crime may be considered spontaneous, typically an impulsive act of passion that is not planned in advance. Legally, the distinction between premeditated and passion-driven crimes, based on the intent and planning involved, affects the charges and sentencing. Therefore, the position of the victim at the time of the attack is very important for analyzing the motive of the crime.

The purpose of this study was to evaluate the biomechanical characteristics of slashing a dummy’s neck using a Chinese kitchen knife while the dummy was in standing and supine positions. We hypothesized that both the victim’s posture and the perpetrator’s gender have a significant influence on 1) kinematic parameters including joint velocity, slashing velocity swing time, and contact time; 2) kinetic parameters including slashing force, relative slashing force, impulse, and energy; and 3) the value of RMS and iEMG of the slashing right upper limb muscles. This study analyzed the kinematic and kinetic characteristics and muscle activation patterns of criminal behaviors when slashing the neck of the victim in different body postures. This work can provide a basis and reference for forensic identification, criminal investigations, and court trials.

## Methods

### Participants

Twenty-four college students, consisting of 12 male and 12 female participants, volunteered to participate in this study. The inclusion criteria were as follows: 1) age between 18 and 28 years; 2) being right-handed; and 3) being in good health, with no history of disease or injury in the past 6 months. This study was approved by the Institutional Review Board. Prior to any data collection, each participant signed a written consent form. Detailed information for all participants is shown in Table 1.

**TABLE 1:** Participant demographic characteristics (mean ± standard deviation).

Gender	Number of participants (N)	Age (years)	Weight (kg)	Height (m)
Male	12	24.2 ± 1.1	73.2 ± 9.1	1.74 ± 0.03
Female	12	23.6 ± 1.2	55.0 ± 5.5	1.63 ± 0.03

### Data collection

The skin of the right upper limb was prepared by shaving off all hair, lightly abrading the skin, and cleaning with alcohol swabs. Electrodes for sEMG were attached over the muscle belly parallel to muscle fibers. The main muscles of the upper limb were evaluated, including the anterior deltoid, medial deltoid, biceps brachii, triceps brachii, extensor carpi radialis longus, flexor carpi ulnaris, and flexor carpi radialis. Manual muscle testing was performed to ensure correct electrode placement. The maximum voluntary contraction (MVC) test was performed according to the “The ABC of EMG” manual (Konrad, 2005).

A total of 35 retro-reflective markers were placed on the participant using the Qualisys PAF Running Package Marker Set, and a six retro-reflective markers were placed on the dummy: bilaterally on the ears and shoulders as well as forehead and sternum. In addition, three retro-reflective markers were placed on the tip of the blade, the hilt of the blade, and between the blade and handle of the knife. A detailed location of the marking point can be found in Li et al. (2022).

**FIGURE 1:**
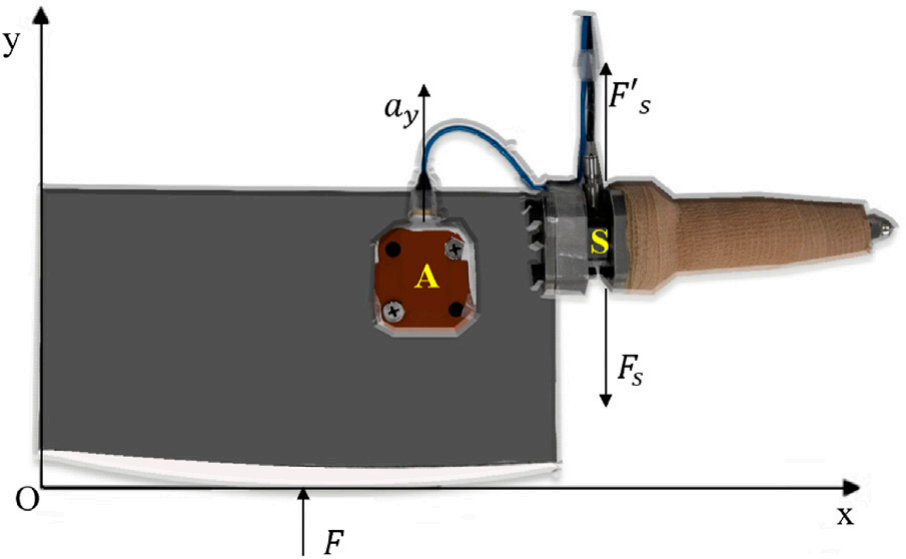
Free body diagram for the instrumented Chinese kitchen knife on the *y*-axis. A indicates the accelerometer with the adapter placed on the COM of the knife blade. S indicates the force sensor with the adapter between the knife handle and the knife blade. $a_{y}$ was the acceleration measured using the accelerometer. $F_{s}$ was the force component along the slashing direction measured using the force sensor, and ${\overset{´}{F}}_{s}$ was the reaction force to $F_{s}$ . F was the reaction force to the slashing force along the direction of slashing, which is equal to the slashing force.

In this study, we utilized a standard Chinese kitchen knife (weight, length, and width were 1.17 kg, 18.3 cm, and 9 cm, respectively), which is the most readily available sharp implement in China. A triaxial force sensor (Kistler 9027c, Switzerland) was mounted by two adapters, which were placed between the knife blade and knife handle (Figure 1). In addition, a small triaxial accelerometer (Kistler 8763b, Switzerland) was attached to the center of mass ( COM ) of the knife blade using an adapter (Figure 1). The dummy was made of PVC silicone material (length and width were 170 cm and 65 cm, respectively). The dummy was placed on a fold-out bed (length, width, and height were 185 cm, 60 cm, and 36 cm, respectively) to simulate the victim being attacked in a supine position.

**FIGURE 2:**
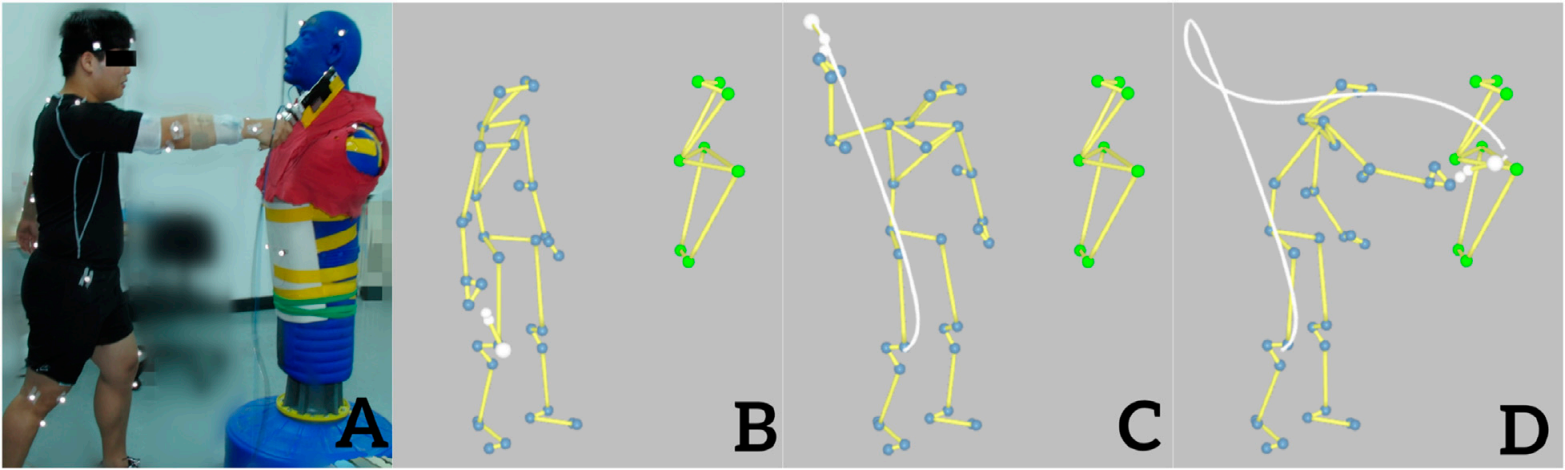
Slashing attack on the neck of a dummy in the standing position **(A)**. Sketch stick diagram of the slashing movement at the moment of start **(B)**, middle **(C)**, and end point **(D)**.

**FIGURE 3:**
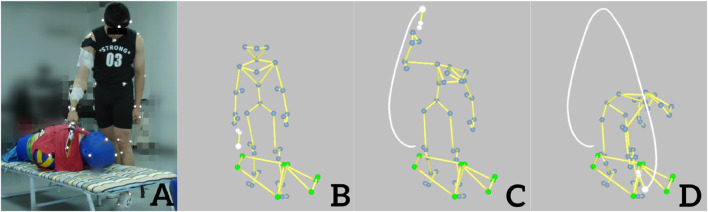
Slashing attack on the neck of a dummy in the supine position in a fold-out bed **(A)**. Sketch stick diagram of the slashing movement at the moment of start **(B)**, middle **(C)**, and end point **(D)**.

The participants held the Chinese kitchen knife and slashed the dummy’s neck while it was in the standing position (Figure 2) and then slashed the dummy’s neck while it was in the supine position (Figure 3), with a 1-min interval between the two postures. To exert the maximal force, the participant was allowed to adjust his/her standing position for the most convenient slashing movement. The three-dimensional (3-D) trajectories of the retro-reflective markers of slashing tasks were recorded using a 3-D motion capture system (Oqus 700, Qualisys Track Manager, Gothenburg, Sweden) with eight cameras at a sample rate of 250 Hz and Qualisys Track Manager software (Version 2018, Qualisys Track Manager, Gothenburg, Sweden). Data from the force sensor and accelerometer were collected using Qualisys Track Manager software, with a sampling frequency of 2,500 Hz. The EMG signals were recorded using an 8-channel wireless acquisition system (Noraxon, Scottsdale, AZ, USA) at a sampling rate of 2,000 Hz. The kinematic, kinetic, and sEMG data collections were synchronized using the Qualisys Track Manager software, and a trigger was used to start all data collection sessions. A detailed description of the kinematic and kinetic data collection can be found in Li et al. (2022). Each participant was asked to complete three acceptable slashing trials with maximum effort with 1-min rest between the trials. An acceptable trial is defined as a trial in which the kinematic, kinetic, and sEMG data were all collected successfully.

### Data processing

Kinematic data were filtered using a fourth-order Butterworth low-pass filter with a cut-off frequency of 25 Hz, and kinetic data were filtered using a second-order Butterworth low-pass filter with a cut-off frequency of 250 Hz.

The slashing movement was divided into a swing phase and a contact phase. The swing phase was defined as the period from the moment the participant began to swing the knife until the moment it reached the dummy. The contact phase was defined as the period from the moment the knife reached the dummy until the moment it left the dummy. The moment the knife reaches the dummy was defined as the time point when the slashing force first exceeded 0 N. The moment the knife left the dummy was defined as the time point when the slashing force dropped below 10 N.

Velocity for the hip, shoulder, elbow, and wrist joint was defined as the maximal velocity for the joint center in the swing phase. Slashing velocity was defined as the velocity of the COM of the instrumented knife at the moment it reached the dummy. The COM position of the instrumented knife was calculated from the mass and coordinates for COM of each accessory, including the knife blade, knife handle, accelerometer, and adapter, as well as the force sensor and two adapters.

Slashing force was defined as the maximal force exerted on the dummy along the slashing direction during the contact phase, which was equal and opposite to F . F was the reaction force of the dummy to the knife along the direction of slashing, and $F_{s}$ was the force component along the slashing direction measured by the force sensor (Figure 1). m was the mass of the knife blade with the accessories (the accelerometer and adapters), and $a_{y}$ was the acceleration along the slashing direction measured by the accelerometer (Figure 1). F was calculated as follows:

$$F-F_{s}=ma_{y},$$

$$F=ma_{y}+F_{s}.$$

The relative slashing force was the slashing force divided by the corresponding body weight (BW). Energy (*E*) was the sum of translational kinetic energy and rotational kinetic energy at the moment when the knife reached the dummy. The impulse was calculated as the force integration during the contact phase. A detailed description of the kinematic and kinetic data reduction can be found in Li et al. (2022).

Raw EMG signals were processed offline using Noraxon MR3.10 software (version 7.13, Cambridge Electronic Design, Cambridge, United Kingdom). The signals were full-wave-rectified and then band-pass-filtered with cut-off frequencies of 10–500 Hz (fourth-order Butterworth band-pass filter) to remove movement artifacts. The root-mean-squared (RMS) value of the corresponding muscle during the swing phase was normalized by dividing RMS over a 20-ms window of the MVC test. IEMG was the integral of EMG during the swing phase and normalized using the MVC test.

### Statistical analysis

To test our hypotheses, a two-way repeated ANOVA was used to test whether the body posture of the victim (standing position/supine position, within-group) and gender (male/female, between groups) of the perpetrator significantly influence the kinematic, kinetic, and muscle activation parameters. All data analyses were performed using version 18.0 of the SPSS computer program package (SPSS, Chicago, IL, USA). Statistical significance was defined as the type I error rate lower than or equal to 0.05.

## Results

The interaction effect between the dummy’s posture and gender was not significant for all kinematic parameters (all *p* > 0.05). There was a main effect for posture on hip and shoulder joint velocities and contact time, and participants showed higher hip joint velocity, smaller shoulder velocity, and shorter contact time when slashing the dummy in the standing position compared to the supine position (all *p* < 0.05; Table 2). Gender also had a main effect on shoulder, elbow and wrist joint velocities, slashing velocity, and contact time, with male participants showing higher velocities for the shoulder, elbow, and wrist joints, increased slashing velocity, and shorter contact times (all *p* < 0.05; Table 2). No significant main effect of gender was detected for hip velocities and swing time (all *p* > 0.05; Table 2).

**TABLE 2:** Kinematic data of slashing on the neck of a dummy in standing and supine positions.

Variable	Male (n = 12)		Female (n = 12)		*p*-value	
	Standing	Supine	Standing	Supine	Gender	Posture
Swing time (s)	1.16 ± 0.23	1.02 ± 0.30	1.22 ± 0.20	1.20 ± 0.21	0.094	0.268
Contact time (ms)	21.23 ± 2.90	24.93 ± 4.04	24.57 ± 4.00	29.87 ± 5.69	0.002^a^	0.001^b^
Hip velocity (m/s)	0.69 ± 0.17	0.56 ± 0.1	0.67 ± 0.19	0.52 ± 0.08	0.508	0.001^b^
Shoulder velocity (m/s)	1.62 ± 0.34	2.21 ± 0.42	1.38 ± 0.38	1.73 ± 0.37	0.017^a^	<0.001^b^
Elbow velocity (m/s)	3.83 ± 0.64	4.02 ± 0.9	3.14 ± 0.63	3.26 ± 0.68	0.012^a^	0.250
Wrist velocity (m/s)	5.00 ± 0.52	5.26 ± 0.85	4.02 ± 0.41	4.23 ± 0.36	<0.001^a^	0.131
Slashing velocity (m/s)	12.11 ± 1.16	11.61 ± 1.63	9.94 ± 1.16	9.86 ± 1.08	<0.001^a^	0.443

^a^ Significant difference between genders of the attacker.

^b^ Significant difference between dummy postures.

It was found that the interaction effect between the dummy’s posture and gender was not significant for all kinetic parameters (all *p* > 0.05). No main effect for the dummy’s posture was detected for slashing force, relative slashing force, impulse, and energy (all *p* > 0.05; Table 3). There was a main effect for gender for slashing force and energy, with higher values for male participants (all *p* < 0.05; Table 3). No main effect for gender was detected for relative slashing force and impulse (all *p* > 0.05; Table 3).

**TABLE 3:** Kinetic data of slashing on the neck of a dummy in standing and supine positions.

Variable	Male (n = 12)		Female (n = 12)		*p*-value	
	Standing	Supine	Standing	Supine	Gender	Posture
Slashing force (N)	912.02 ± 241.12	958.55 ± 289.37	775.57 ± 200.27	733.09 ± 173.23	0.034^a^	0.968
Relative slashing force (BW)	12.67 ± 3.97	13.40 ± 4.94	14.16 ± 3.54	13.38 ± 3.13	0.604	0.977
Impulse (Ns)	9.31 ± 2.65	10.01 ± 2.93	8.31 ± 1.91	8.93 ± 1.56	0.228	0.158
Energy (J)	37.29 ± 7.51	36.17 ± 12.34	24.91 ± 4.69	25.37 ± 5.63	<0.001^a^	0.890

^a^ Significant difference between genders of the attacker.

^b^ Significant difference between dummy postures.

The interaction effect between the dummy’s posture and gender was not significant for all sEMG data (all *p* > 0.05). There was a main effect of posture for RMS of the anterior deltoid and iEMG of the anterior deltoid (all *p* < 0.05; Table 4), with higher values for the standing dummy. No main effect for the dummy’s posture was detected for other sEMG data (all *p* > 0.05; Table 4). There was a main effect of gender for RMS and iEMG of the anterior deltoid, biceps brachii, extensor carpi radialis longus, and flexor carpi ulnaris (all *p* < 0.05; Table 4), with higher values found for female participants. No main effect for gender was detected for the medial deltoid, triceps brachii, and flexor carpi radialis (all *p* > 0.05; Table 4).

**TABLE 4:** sEMG data of slashing on the neck of a dummy in standing and supine positions.

Variable (% MVC)		Male (n = 12)		Female (n = 12)		*p*-value	
		Standing	Supine	Standing	Supine	Gender	Posture
RMS	Anterior deltoid	26.08 ± 9.76	17.86 ± 8.36	53.69 ± 25.62	34.29 ± 19.27	0.004^a^	<0.001^b^
	Medial deltoid	22.11 ± 9.02	23.11 ± 12.28	29.04 ± 12.64	26.04 ± 12.37	0.279	0.522
	Biceps brachii	17.12 ± 6.26	19.51 ± 6.86	27.95 ± 9.07	25.63 ± 9.62	0.014^a^	0.972
	Triceps brachii	34.10 ± 25.17	29.76 ± 14.97	33.78 ± 13.97	30.39 ± 14.89	0.840	0.219
	Extensor carpi radialis longus	27.63 ± 14.87	32.84 ± 17.51	50.94 ± 22.65	48.25 ± 22.10	0.009^a^	0.507
	Flexor carpi ulnaris	33.45 ± 21.53	34.48 ± 21.64	47.83 ± 14.90	42.70 ± 14.95	0.015^a^	0.476
	Flexor carpi radialis	34.12 ± 24.71	37.34 ± 29.73	41.14 ± 13.32	35.24 ± 7.13	0.446	0.467
IEMG	Anterior deltoid	35.01 ± 13.09	23.48 ± 11.88	76.36 ± 39.25	49.82 ± 29.82	0.002^a^	<0.001^b^
	Medial deltoid	29.02 ± 10.82	28.50 ± 14.64	41.15 ± 19.74	38.45 ± 20.55	0.099	0.510
	Biceps brachii	22.82 ± 8.81	24.65 ± 7.90	38.97 ± 12.16	36.48 ± 11.37	0.002^a^	0.815
	Triceps brachii	46.46 ± 39.00	38.78 ± 20.40	46.58 ± 17.86	44.68 ± 23.73	0.583	0.753
	Extensor carpi radialis longus	36.20 ± 16.92	42.86 ± 24.60	70.76 ± 27.29	70.65 ± 33.20	0.003^a^	0.335
	Flexor carpi ulnaris	42.94 ± 22.12	43.77 ± 31.02	66.64 ± 20.33	61.44 ± 20.82	0.016^a^	0.454
	Flexor carpi radialis	46.50 ± 34.02	45.69 ± 32.15	57.44 ± 17.68	50.85 ± 9.94	0.223	0.215

^a^ Significant difference between genders of the attacker.

^b^ Significant difference between dummy postures.

## Discussion

The findings of this study provide a theoretical foundation for law enforcement and forensic experts regarding the quantitative assessment of slashing attacks using a Chinese kitchen knife and other types of slashing knives. The integration of quantitative biomechanical data solidifies the evidentiary basis for the adjudication and trial processes in cases involving slashing attacks. The results partially support our first hypothesis that both the victim’s posture and the perpetrator’s gender have a significant influence on joint velocity, slashing velocity, swing time, and contact time. This study indicated that slashing the standing dummy’s neck resulted in higher hip velocity, smaller shoulder velocity, and shorter contact time compared to slashing it in the supine position. Male participants showed higher values of shoulder, elbow, and wrist joint velocities, higher slashing velocity, and shorter contact time than female participants.

Our previous research found that shoulder, elbow, and wrist joint velocities and slashing velocity showed higher values when slashing the chest than when slashing the neck (Li et al., 2022), and the difference may be caused by the difference in height of the body parts being attacked. However, in this study, there was no significant difference in shoulder, elbow, and wrist joint velocities and slashing velocity between different body postures when the dummy was attacked. The discrepancies in participants’ hip and shoulder velocities when slashing the dummy in its different body postures can probably be attributed to different action patterns. When slashing the standing dummy, the slashing target is in the front, and the participant would mainly bend the trunk and lower limbs around the vertical axis in the anterior–posterior direction to perform the slashing action. When slashing the dummy in its supine position, the slashing target is low, so he/she would mainly rotate the trunk around the coronal axis, and the hip rotation is minimal. Therefore, hip velocity was higher when participants slashed the dummy’s neck while it was in the standing position. When slashing the dummy in its supine position, the kitchen knife moves downward, and the gravity of the knife helps the movement. Theoretically, the resistance that the participant needs to overcome would be lower when the dummy is in the supine position. Therefore, shoulder joint velocity was higher when participants slashed the neck of the supine dummy. Nevertheless, although there were significant differences in shoulder and hip velocities, there was no significant difference in elbow and wrist joint velocities and slashing velocity. The reason may be that the slashing target around the neck is relatively narrow, especially while the dummy is in the supine position. In addition, the Chinese kitchen knife is relatively large, and in order to ensure the accuracy of slashing, the participant needs to make certain adjustments to the knife, which may affect the slashing action and result in changes in slashing velocity. The results of our study indicate that when a male perpetrator wields a knife, he can produce greater knife velocity than a female perpetrator, which is consistent with previous studies (Bleetman et al., 2003; Trinh et al., 2017). Trinh et al. (2017) showed that male participants achieved higher striking velocities when using steel rods than female participants. Slashing velocity is key when assessing the possibility or the degree of penetration into the skin tissue and can also be used to estimate the slash force to further assess the risk of injury and death. Our study quantified the slashing velocity of using a Chinese kitchen knife to slash the dummy’ neck while it was in standing and supine positions, and the results showed average slashing velocities of 12.1 m/s and 11.6 m/s for male participants and 9.9 m/s and 9.9 m/s for female participants, respectively. The slashing velocity by male participants was 17.7%–21.8% faster than that by female participants, which may result in more severe damage.

Neither the gender of the participant nor the body posture of the dummy being attacked had a significant effect on swing time, which is partly due to the fact that this study did not give specific instructions to the participants on how to perform the slashing attack, with the purpose to more realistically replicate the actual crime process. Therefore, some participants may pause slightly when raising the knife above the shoulder, which results in no significant difference in swing time. Our study quantified the swing time needed to slash the dummy’s neck while it was in standing and supine positions, and the results showed average velocities of 1.16 and 1.22 s for male and female participants in the standing position and 1.02 and 1.20 s for male and female participants in the supine position, respectively. The swing phase begins when the perpetrator starts to swing the knife and is the process throughout which the victim is being harmed. A recent study examined the performance of four stationary knife attacks to determine how long it takes to execute each motion, and the results showed that the time it takes to execute knife stab (thrust and overhead) and slash (Figure 8 type and reverse) were 0.61, 0.68, 1.07, and 0.62 s, respectively (Kantor et al., 2022), which is faster than the swing time in our study. This is likely due to the weight of the weapon being used and the different modalities for attack. Our study utilized an instrumented kitchen knife that weighs 1.17 kg, which is much heavier than the knife (0.19 pounds) used in the abovementioned study. In addition, our study required participants to slash into the dummy’s neck, while the abovementioned study only measured the performance time without a target. Another previous study showed that the average time for a human being to respond to visual stimuli is approximately 0.15–0.46 s (Sanchez et al., 2017). The results of our study, combined with those of previous studies, indicate that if the victim keeps an appropriate distance from the perpetrator and is vigilant enough to detect danger in time, the victim may be able to avoid the corresponding slashing injury from a Chinese kitchen knife or other comparative knives.

The contact time of slashing the standing dummy’s neck was shorter than that in the supine position. The significant difference in contact time between the dummy’s postures may be due to the different slashing action modes, especially the movement of the upper limbs and trunk. The trunk is erect, with the upper limbs being in a similar horizontal position when slashing the dummy in the standing position. The trunk is bent over with upper limbs pointing down when slashing the dummy in its supine position, and comparatively speaking, slashing in its standing position makes it easier to get the knife back. In this study, we found that the contact time for male participants was smaller than that for female participants, which may be because male participants are generally stronger than female participants, so it is faster to move the knife away from the dummy.

The results did not support our second hypothesis, which stated that both the victim’s posture and the perpetrator’s gender have a significant influence on slashing force, relative slashing force, impulse, and energy. There are only significant differences in slashing force and energy between genders and no difference in kinetic parameters (force, impulse, and energy) between the different postures of the dummy attacked. Our previous research found higher energy when slashing the chest than when slashing the neck (Li et al., 2022), which is inconsistent with this study. As previously mentioned, the participant may adjust the slashing angle and velocity of the Chinese kitchen knife to ensure the accuracy of the slashing, which may affect the kinetic parameters.

In this study, we found that the slashing force of male participants (912.0 N for the standing dummy and 958.6 N for the supine dummy) was 17.6%–30.8% higher than that of female participants (775.6 N for the standing dummy and 733.1 N for the supine dummy). Many previous studies focused on the effects of stabbing force on penetrating soft tissues, bones, and puncture-resistant materials. O'Callaghana et al. (2001) assessed the resistance force when penetrating skin, subcutaneous fat, and skeletal muscle measured using a pressure sensor, and the resistance force was 55 N, 20 N, and 40 N, respectively. Four commonly available household knives with different geometries were used to pierce synthetic materials simulating skin, fat, and cartilage, and the results showed that the maximum penetration force was no more than 40 N (Gilchrist et al., 2008). Another study indicated that the peak force of the rib fractures caused by the blunt and sharp weapon was 733 N and 392 N, respectively (Gaudet et al., 2020). Although the slashing force of male participants was greater than that of female participants, there was no significant difference in the relative slashing force between genders after normalizing for body weight. This suggests that female participants with large body weight could also cause high slashing force similar to that of male participants with the same weight. Therefore, at the stage of investigating suspects, the possibility of women committing crimes cannot be ruled out solely based on the extent of the injury.

In this study, we found that the slashing energy of male participants (37.3 J for the standing dummy and 36.2 J for the supine dummy) was 42.6%–65.4% higher than that of female participants (24.9 J for the standing dummy and 25.4 J for the supine dummy). Energy is a key indicator to measure the extent of human injury caused by weapons and has always been a hot topic in the research of sharp force injury. A study showed that the energy needed to pierce synthetic materials was no more than 1 J (Gilchrist et al., 2008). When blowing with a rod, the energy threshold to cause fracture in sheep bones and bionic bones is approximately 22 J (Gentile et al., 2019). Chadwick et al. (1999) showed that anti-puncture materials need to withstand approximately 36 J. In addition, there was no significant difference in slashing force, relative slashing force, energy, and impulse between the two postures of the dummy, suggesting that the damage of slashing the neck for standing and supine victims may not be significantly different. However, the neck position is relatively narrow, and it is difficult to slash. Therefore, the results of this study may not be applicable to other body parts being slashed, and further studies should be conducted on slashing the chest, head, and other parts with different body postures.

The results of our study indicate that female participants exhibit greater muscle activation than male participants. The RMS and iEMG of the anterior deltoid, biceps brachii, extensor carpi radialis longus, and flexor carpi ulnaris of female participants are higher than those of male participants, and the RMS and iEMG of the anterior deltoid are higher when slashing a standing dummy. This is inconsistent with our third hypothesis. RMS and iEMG are important indicators for muscle activation. There was one study that recruited 10 professional deboners to perform the same carving task with both very sharp and very dull knives, and the results showed that better blade sharpness leads to significantly lower EMGs for the flexor digitorum superficialis, biceps brachii, triceps brachii, anterior deltoid, and upper trapezius muscles (Claudon and Marsot, 2006). A previous study implied that the more difficult a task is to complete, the higher the degree of muscle activation (Claudon and Marsot, 2006). The instrumented knife used in our study is comparatively heavy and is, therefore, more difficult for female participants with lower muscle strength to lift and swing; this difficulty may result in higher values of RMS and iEMG for some upper limb muscles. In addition, slashing movement is a classic upper limb whiplash movement, with a sequential acceleration and brake proximally to distally, and the muscles around the shoulder joint are activated first. Therefore, the activation and recruitment of the anterior deltoid may be different between slashing the dummy in standing and supine positions.

In this study, we utilized biomechanical methods to evaluate the kinematic, kinetic, and sEMG characteristics of using a Chinese kitchen knife to slash the dummy’s neck while it was in different body postures (standing and supine). Overall, there was a significant difference in the slashing action mode between postures, showing different joint velocities (hip and shoulder) and different activation levels of deltoid exertion. However, there was no difference in slashing speed, slashing force, and energy, which are key metrics associated with injury severity. Our previous study found that the slashing distance and space required for knife slashing were different between the two postures (Yuan et al., 2022). Combined with this study, it could be inferred that when identifying the victim’s posture being attacked, the spatiotemporal indicators of the slashing behavior and the measures of the wound should be considered. Compared to the victim’s posture, the gender of the attacker may have a greater effect on slashing severity, and the gender difference may partly be due to the differences in body weight, height, and strength.

Quantitative biomechanical data from this study could provide auxiliary clues for investigators to determine criminal behavior (crime of passion or premeditated crime) and provide important information for criminal investigations and court trials. There are limitations to this study. First, for safety reasons, the slashing target of our study was a dummy, which may not be able to fully imitate the actions of the actual victim. Therefore, there may be a slight difference compared to an actual slashing scene. Furthermore, this study did not study the characteristics of the wound, which is of great significance for determining the direction of the cut. Further studies that combine wound characteristics and evasive actions are warranted. Finally, the study focused on exclusively young, healthy adults and may not fully account for the observed patterns of slashing behavior. Further studies may expand the research to include participants of various ages to enhance our comprehension.

## Conclusion

There was no difference in slashing speed, slashing force, and energy when using a Chinese kitchen knife to slash the dummy’s neck while it was in different body postures (standing and supine), suggesting that the severity of injury may not be different. However, there were significant differences in slashing action patterns between two body postures, with longer contact time, smaller hip velocity, greater shoulder velocity, and less muscle activation level of deltoid exertion when slashing the dummy’s neck in the supine position. Compared to body postures, gender may have a greater effect on slashing severity, and gender differences may be partly related to the body weight difference. The findings of this study may provide quantitative indicators and references for case reconstruction and court’s conviction and sentencing.

## Data Availability

The original contributions presented in the study are included in the article/Supplementary Material; further inquiries can be directed to the corresponding author.
